# Efficacy of EWINDOW for prevention of delirium at intensive care units: A protocol for systematic review and meta-analysis

**DOI:** 10.1097/MD.0000000000028598

**Published:** 2022-01-21

**Authors:** Jijie Liu, Jie Wang

**Affiliations:** Medical Faculty, Yunnan University of Business Management, Kunming, Yunnan, PR China.

**Keywords:** delirium, EWINDOW, intensive care units

## Abstract

**Background:**

Critically ill patients in intensive care units (ICUs) have restricted exposure to natural light throughout the day. As a result, they are more prone to undergo disruptions in circadian rhythmicity and sleep patterns, which may contribute to the development of delirium. As a measure to synchronize to the patterns of natural sunlight, EWINDOW has been proved to prevent delirium in some studies. Hence, the aim of this systematic review to evaluate the effectiveness of EWINDOW so as to prevent delirium.

**Methods:**

From January 2012 to December 2021, electronic databases such as China National Knowledge Infrastructure Database, Excerpta Medica database, PubMed, Cochrane Central Register of Controlled Trials, Wan Fang, and Cumulative Index of Nursing and Allied Health Literature are aimed to look for appropriate randomized controlled trials to evaluate the therapeutic impact of EWINDOW on delirium prevention. To be more exact, related studies are chosen, data is retrieved and the risk of bias is examined, then a meta-analysis is carried out in order.

**Results:**

The goal of this study is to show how effective EWINDOW is at preventing delirium in ICU. The incidence and duration of delirium are included in our outcome measures.

**Conclusions:**

This review evaluates related studies on the therapeutic effects of EWINDOW on the prevention of delirium in the ICUs.

**Dissemination and ethics:**

The findings of this study will be published in a peer-reviewed journal. As the study relies on publicly available data, no ethical approval is required. Furthermore, throughout the data analysis procedure, anonymity is protected.

OSF Registration: https://osf.io/NP3EW/

## 1 Introduction

Delirium is an organically caused acute confusional state marked by erratic behavior, attention impairments, and extreme disorganization.^[1]^ Meanwhile, it may affect 60% to 80% of mechanically ventilated and 20% to 50% of nonmechanically ventilated critically ill patients admitted to the intensive care units (ICU).^[2]^ ICU delirium is linked to longer hospital stays,^[3]^ increased risk of mortality,^[4]^ and long-term cognitive impairment.^[5]^ Delirium is linked to more disruption of the circadian sleep-cycle and increased daytime sleep.^[6]^ In this scenario, patients in the ICU who have insufficient access to sunlight throughout the day are at a higher risk of establishing circadian rhythmicity and sleep patterns, which may contribute to the development of delirium.^[7]^ This is why clinical practice guidelines advocate a multicomponent, nonpharmacologic regimen for the prevention and management of pain, agitation/sedation, delirium, immobility, and sleep disruption, which includes a sleep-focused intervention.

EWINDOW is a brand new lighting product, which patented design integrates a 3 dimensional simulated sky scene, synchronized to the patterns of natural sunlight throughout the day, adjusting for seasons, latitude, and ambient light conditions to emulate an exterior window. It perfectly integrates the latest light emitting diode technology, optical design, and intelligent control systems. Special light emitting diodes are used to light up the sky and clouds, then transform them into a translucent 3 dimensional image, so the performance of the EWINDOW simulates a real sky and clouds. Settings are easily adjusted using a wall mounted control panel, smartphone app, or digital addressable lighting interface controls.^[8]^ The previous study has proved that multi-component nonpharmacologic interventions included simulated natural sunlight during the day (similar with EWINDOW) could reduce the incidence of delirium,^[9]^ but other studies have shown no effect on either delirium incidence or ICU length of stay.^[10,11]^

As a consequence, the current review's purpose is to comprehensively investigate all randomized controlled trials (RCTs) to assess the efficacy of EWINDOW in preventing delirium in critically ill patients.

## 2 Materials and methods

On December 14, 2021(Registration: https://osf.io/NP3EW/), On Open Science Framework the current comprehensive literature protocol has been registered. The Preferred Reporting Items for Systematic Reviews, Meta-Analysis Protocol guidelines^[12]^ and the Cochrane Handbook for Systematic Reviews of Interventions were used to create it. Any changes to the present review must be documented.

## 3 Criteria for study inclusion

### 3.1 Type of RCTs study

The present study will include RCTs that are connected to the use of EWINDOW and are important to the prevention of delirium in critical situations, with no constraints on publication status or language. Furthermore, this review excludes quasi-RCTs, non-RCTs, case series, case reports, uncontrolled trials, crossover articles, and laboratory studies. In addition, non-RCTs, quasi-RCTs, case series, case reports, uncontrolled trials, crossover papers, and laboratory research are not included in this study.

### 3.2 Subject type

Subjects which receive mechanical ventilation for more than 48 hours in the ICU were gathered regardless of the age, gender, or race.

### 3.3 Intervention type

EWINDOW, which replicated natural sunshine during the day, was one of the interventions used in this study. The Control is achieved through standard care. All patients in both the intervention and control groups should receive the same regular delirium care at ICUs, according to the delirium care package.

### 3.4 Type of outcome metric

#### 3.4.1 Primary endpoint (s)

The primary endpoint will be the occurrence and duration of delirium.

#### 3.4.2 Supplementary endpoint (s)

The lengths of time spent in the ICU and in the hospital are the secondary endpoints.

## 4 Techniques of study retrieval and identification

### 4.1 Electronic databases

The following electronic databases are used to identify related RCTs:

- China National Knowledge Infrastructure database, from 2012 to present;
- Cochrane Central Register of Controlled Trials, from 2012 to present;
- Cumulative Index of Nursing and Allied Health Literature, from 2012 to present;
- Excerpta Medica database (from 2011 to present);
- Wan Fang database (from 2012 to present);
- Ovid MEDLINE ALL (from 2012 to present).
- PubMed Database (from 2012 to present);

Moreover, Clinical trial databases, such as the Netherlands National Trial Register, the Chinese Clinical Trial Registry, and ClinicalTrials.gov, are also searched to find those trials that are still underway but have not yet been published.

Language is not a factor in the screening of RCTs.

### 4.2 Data collection and analysis

#### 4.2.1 Relevant study selection

EndNote X9 is used to manage records from the electronic databases that were searched. The names of the studies and their abstracts will be chosen with care. After that, 2 reviewers (JW and JL) will look over the entire text of connected publications to see if they meet our inclusion criteria. Following that, these 2 reviewers will look over the relevant research to see if they meet the requirements, and any disagreements will be addressed by the opinion of a third reviewer. Our selection approach is followed for selecting studies, and the results are reflected in the Preferred Reporting Items for Systematic Reviews, Meta-Analysis flow chart. After that, the suggestions will be assessed using the Grading of Recommendations Assessment, Development, and Evaluation method.

#### 4.2.2 Data extraction and analysis

To collect data, we will develop a typical data extraction form. Two reviewers obtained the following information (JW and JL):

- Data source: identify study, publication time, first author, and title;
- Attendees: Inclusion and exclusion criteria for the study;
- Study method: design of study, study sample size, concealment of allocation randomization, blinding, insufficient data or selective report, extras bias sources;
- Intervention: Types of intervention;
- Control method: types of control;
- Consequence: Outcome measures considered.

#### 4.2.3 Assessment of the risk of bias

Two independent reviewers (JW and JL) separately assess the potential bias among some of the included studies using the Cochrane Risk of Bias Tool, and any disputes will be resolved through mutual negotiation or a third reviewer reaching an agreement. Each conclusion is produced and expressed in a Risk of Bias graph, which is then paired with sensitivity analysis to evaluate the review results. To be more specific, the risk of bias in each area is graded as insufficient, adequate, or unclear. In this review, the possibility of heterogeneity in sensitivity analysis is investigated by looking at the concealment of allocation. Blinding extent (where necessary), noncompliance, loss to follow-up, uniformity of outcome assessment, and use of intention-to-treat analysis are all included in the Risk of Bias, which summarizes all registered articles.

#### 4.2.4 Study data analysis

Related Stata software is used to conduct meta-analyses (15.1). To compare continuous variables, weighted mean difference is utilized, and the cumulative statistical effects of the 2 are then integrated. The Chi-square test is used to analyze potential heterogeneity in each of the enrolled research topics, with *I*^2^ > 50% indicating significant judgment and the use of a random effect model; otherwise, *I*^2^ ≤ 50% implies that the enrolled studies are homogeneous and that a fixed effect model was used. In contrast, effect size will be reported as a 95% confidence interval, with a *P* = .05 difference suggesting statistical significance.

When there is at least 1 outlier study with results that differ from the rest, a sensitivity analysis was undertaken to assess whether there is any heterogeneity and to rule out the outliers. An examination of sensitivity will also be performed to look at the impact of trial quality on effect estimates. Allocation concealment, a sufficient number of allocation sequences are created, and (intention-to-treat) analysis is used for all excellent components of the methodology. When there is enough data, a meta-regression analysis is performed.

#### 4.2.5 Bias of publication

When there are enough trials recovered (>10), funnel plots (effect size as a function of standard error) are to be constructed to look at the issue of publication bias.

#### 4.2.6 Ethical statement and dissemination

Because all of the data in this study was collected from published journals, ethical approval was not required.

## Author contributions

**Conceptualization:** Jijie Liu, Jie Wang.

**Data curation:** Jijie Liu, Jie Wang.

**Formal analysis:** Jijie Liu, Jie Wang.

**Funding acquisition:** Jijie Liu, Jie Wang.

**Investigation:** Jijie Liu, Jie Wang.

**Methodology:** Jijie Liu, Jie Wang.

**Project administration:** Jie Wang.

**Resources:** Jijie Liu, Jie Wang.

**Software:** Jijie Liu.

**Supervision:** Jie Wang.

**Validation:** Jijie Liu.

**Visualization:** Jie Wang.

**Writing – original draft:** Jijie Liu.

**Writing – review & editing:** Jie Wang.

## References

[1] American Psychiatric Association. Diagnostic and Statistical Manual of Mental Disorders. 5th ed. Washington, DC: American Psychiatric Association; 2013.

[2] Ely EW Inouye SK Bernard GR. Delirium in mechanically ventilated patients: validity and reliability of the confusion assessment method for the intensive care unit (CAM-ICU). JAMA 2001;286:2703–10. 11730446 10.1001/jama.286.21.2703

[3] Thomason JW Shintani A Peterson JF. Intensive care unit delirium is an independent predictor of longer hospital stay: a prospective analysis of 261 non-ventilated patients. Crit Care 2005;9:R375–81. 16137350 10.1186/cc3729 PMC1269454

[4] van den Boogaard M Peters SA van der Hoeven JG. The impact of delirium on the prediction of in-hospital mortality in intensive care patients. Crit Care 2010;14:01–5. 10.1186/cc9214 PMC2945129 20682037

[5] Pandharipande PP Girard TD Jackson JC. Long-term cognitive impairment after critical illness. N Engl J Med 2013;369:1306–16. 24088092 10.1056/NEJMoa1301372 PMC3922401

[6] Devlin JW Skrobik Y Gélinas C. Clinical practice guidelines for the prevention and management of pain, agitation/sedation, delirium, immobility, and sleep disruption in adult patients in the ICU. Crit Care Med 2018;46:e825–73. 30113379 10.1097/CCM.0000000000003299

[7] Weiss B Spies C Piazena H. Exposure to light and darkness and its influence on physiological measures of intensive care unit patients-a systematic literature review. Physiol Meas 2016;37:R73–87. 27510570 10.1088/0967-3334/37/9/R73

[8] Brightway LED Lighting, 2021. EWINDOW - Brightway LED Lighting. 2021. Available at: https://www.brightwayledlighting.com/ewindow. Accessed December 21, 2014.

[9] Colombo R Corona A Praga F. A reorientation strategy for reducing delirium in the critically ill. Results of an interventional study. Minerva Anestesiol 2012;78:1026–33. 22772860

[10] Ono H Taguchi T Kido Y Fujino Y Doki Y. The usefulness of bright light therapy for patients after oesophagectomy. Intensive Crit Care Nurs 2011;27:158–66. 21511473 10.1016/j.iccn.2011.03.003

[11] Taguchi T Yano M Kido Y. Influence of bright light therapy on postoperative patients: a pilot study. Intensive Crit Care Nurs 2007;23:289–97. 17692522 10.1016/j.iccn.2007.04.004

[12] Shamseer L Moher D Clarke M. Preferred reporting items for systematic review and meta-analysis protocols (PRISMA-P) 2015: elaboration and explanation. BMJ 2015;350:01–25. 10.1136/bmj.g7647 25555855

